# Impact of Medications and Marijuana Use on Hyposalivation and Xerostomia in Adults

**DOI:** 10.3390/ijerph22111700

**Published:** 2025-11-11

**Authors:** Carter Gehlken, Moni Ahmadian, Neamat Hassan Abubakr

**Affiliations:** 1 School of Dental Medicine, University of Nevada Las Vegas, Las Vegas, NV 89106, USA; gehlken@unlv.nevada.edu; 2 Department of Clinical Sciences, School of Dental Medicine, University of Nevada Las Vegas, Las Vegas, NV 89106, USA; moni.ahmadian@unlv.edu; 3 Department of Biomedical Sciences, School of Dental Medicine, University of Nevada Las Vegas, Las Vegas, NV 89106, USA

**Keywords:** hyposalivation, dry mouth, xerostomia, recreational drug

## Abstract

Background/Objectives: Hyposalivation is a prevalent yet underrecognized factor contributing to oral health deterioration, often influenced by systemic disease, medication use, and recreational drug exposure. With rising use of mental health and cardiovascular medications, as well as increasing marijuana use among younger populations, there is a need to assess real-world data on xerostomia and hyposalivation prevalence and associated risk factors. This study aimed to evaluate the prevalence of hyposalivation and xerostomia, and its etiological associations among adult patients at the University of Nevada, Las Vegas (UNLV) School of Dental Medicine Clinics. Methods: A retrospective cohort study was conducted using electronic health record (EHR) data from 1600 randomly selected patients aged 30 years and older, treated between 1 January 2014, and 31 May 2023. Data on demographics, medical and social history, medication use, and oral health status were extracted. Hyposalivation was identified via chart review, and multivariate logistic regression was used to analyze associated risk factors. Results: Hyposalivation and xerostomia were identified in 705 patients (44.06%). Marijuana use was the strongest independent predictor across all age groups (RR = 3.10, *p* < 0.05). Among patients aged 30–35, use of antihypertensive (OR = 3.05, *p* < 0.05) and mental health medications (OR = 1.81, *p* < 0.05) were significantly associated with hyposalivation. A strong correlation was also found between hyposalivation and elevated caries risk (χ^2^ = 205.99, *p* < 0.001). Conclusions: Hyposalivation and xerostomia are increasingly observed in younger adults, linked to pharmacological and behavioral factors.

## 1. Introduction

Hyposalivation represents a significant and often underrecognized contributor to oral health decline both globally and in the United States. Despite increasing awareness of this clinical sign, dental treatment outcomes may be adversely impacted if salivary flow is not appropriately considered—particularly in patients with identifiable risk factors such as medication use and recreational drug exposure. The growing prevalence of such risk factors in the American population, coinciding with rising rates of cardiovascular and mental health disorders and the legalization of cannabis in many U.S. states, underscores the importance of evaluating real-world data to guide clinical decision-making. The distinction between xerostomia and hyposalivation lies in the parameter being assessed, subjective patient perception versus objective glandular function. Subjective xerostomia: the patient’s perception of having a dry mouth, which is determined through patient self-report. In contrast, objective hyposalivation denotes a quantifiable reduction in salivary flow rate, determined by sialometric assessment of unstimulated and/or stimulated whole saliva [1].

The implications of reduced salivary flow are well documented, given saliva’s protective roles in buffering, lubrication, and microbial control. These functions are critical in maintaining oral health, and reductions in salivary output are associated with increased risk of caries, periodontitis, candidiasis, and compromised prosthetic retention [2,3]. There is also considerable risk in failure of direct and indirect restoration in patients with compromised salivary flow [4].

Notably, many of the existing studies focus primarily on older or institutionalized populations [3,5,6]. While age has traditionally been considered a contributing factor, recent evidence suggests that hyposalivation may be more attributable to polypharmacy, systemic disease, and the use of certain drug classes, rather than aging itself [3,7,8,9,10]. Other studies focusing on histopathology of xerostomia show that much of the age-related changes seen in salivary glands of older patients are due to autonomic innervation rather than salivary gland atrophy, suggesting an outside contributor plays more of a role than the process of aging itself [11]. Medications such as antidepressants, antipsychotics, bronchodilators, antihistamines, and anticholinergics are commonly implicated [12,13,14,15], and the cumulative effects of multiple prescriptions further amplify risk in elderly and medically complex individuals [7,13,15,16].

Emerging data also demonstrates the growing impact of hyposalivation in younger populations. Several studies highlight the role of mental health medications, particularly Selective Serotonin Reuptake Inhibitors (SSRIs) and Tricyclic antidepressants (TCAs), in reducing salivary flow by as much as 30% to 60% [17,18]. Additionally, the increasing prevalence of cannabis and e-cigarette use among adolescents and young adults is associated with a higher frequency of dry mouth symptoms [19,20,21,22,23]. These trends signal a shift in the xerostomia risk profile that includes not only the elderly, but also a significant proportion of younger individuals affected by modern pharmacological and social behaviors.

Despite this body of research, few studies have comprehensively analyzed hyposalivation and xerostomia risk factors across a broad patient population in a real-world clinical setting. Retrospective evaluations using electronic health record (EHR) data provide an opportunity to assess trends in dry mouth prevalence and associated risk variables, such as age, medication usage, and lifestyle factors, within diverse clinical populations. EHR-based studies are particularly valuable in identifying clinical patterns that may not be captured in controlled trial settings, offering meaningful insights into oral health outcomes.

The present retrospective study aims to evaluate the prevalence of hyposalivation and xerostomia among patients seen at the University of Nevada, Las Vegas (UNLV) School of Dental Medicine Clinics and to identify associated etiological factors contributing to its occurrence. The primary research question addresses the prevalence of xerostomia within this patient population and examines potential correlations between self-reported xerostomia and contributory etiological factors. By examining these variables associated with dry mouth symptoms, this study seeks to improve prediction and management strategies in clinical dental care.

## 2. Materials and Methods

This retrospective cohort study was conducted at the UNLV Dental Medicine Clinics, a public outpatient dental care facility located in Clark County, Nevada. The study protocol was reviewed and approved by the UNLV Institutional Review Board and was carried out in accordance with all applicable ethical guidelines and patient confidentiality regulations. All clinical photographs were utilized with consent of the patient.

Patient records were reviewed for the period between 1 January 2014, and 31 May 2023. Inclusion criteria required patients to be aged 30 years or older and to have both a completed medical history and a Caries Risk Assessment updated within the previous 12 months. Patients were excluded if their records were incomplete, duplicated, they failed to return for follow-up, or inadequate clinical data was present for clinicians to determine presence of hyposalivation. Among 12,067 eligible patients, 1600 patient records were simply randomly sampled (margin of error of 2.5% with 95% confidence), and their EHRs analyzed in this study.

Clinical and demographic data were extracted from the UNLV School of Dental Medicine’s EHR system (axiUm) using a filtered search by patient age (≥30 years). Extracted variables included demographic characteristics (age, sex, and ethnicity), relevant medical conditions (such as Type II Diabetes, Hypertension, Hyperlipidemia and Anxiety), social history (such as nicotine, recreational drug, and alcohol use), and prescribed medications. Prescription medications were only considered if taken by mouth at least once daily. No distinction was made between recreational and medical marijuana. Nicotine, marijuana, and alcohol usage was defined as using the drug at least one time per week as reported by the patient. All data were fully de-identified prior to analysis and were stored in a secure, password-protected research database accessible only to authorized members of the study team.

The primary outcome assessed was the presence and degree of hyposalivation and xerostomia, identified through a manual review of electronic dental records. Hyposalivation was documented based on quantitative clinical measurements of both stimulated and unstimulated salivary flow rates recorded during clinical examinations, rather than as a simple binary (yes/no) variable. Xerostomia, on the other hand, was identified as a subjective symptom reported by patients during the same encounters. Secondary outcomes included caries risk classification (categorized as low, moderate, or high) according to the American Dental Association Caries Risk Assessment Form, and overall dental status as measured by the number of decayed, missing, and filled teeth (DMFT index).

As no additional examinations were performed specifically for this study and all data were analyzed and presented anonymously, this study was granted exempt status by the Institutional Review Board (IRB) of the University of Nevada, Las Vegas (UNLV; #UNLV-2023-479). All patients provide general consent during their initial clinic visit for the use of de-identified data in research. The study adhered to the principles of Good Clinical Practice and complied with the ethical standards outlined in the World Medical Association (WMA) Declaration of Helsinki (1975), as revised in 2013.

Potential contributing risk factors were summarized as percentages to facilitate comparison and detect overall trends. Subsequently, multivariate logistic regression analysis was used to assess associations between these risk factors and hyposalivation when variables could be appropriately converted into binary or multi-binary responses. Chi-squared tests were used when categorical data could not appropriately be analyzed using logistic regression. Relative risk calculations were derived from marginal predictions and calculated manually following output from SPSS software (Version 28.0). Multicollinearity and potential interactions among variables were not assessed in the present investigation due to the limited sample size and to preserve the model’s ability to estimate the independent contribution of each risk factor. Statistical significance was defined as a *p*-value of <0.05. All statistical calculations were performed using IBM SPSS software (Version 28.0).

## 3. Results

Among 12,067 eligible subjects, 1600 were sampled. Of these, 705 patients (44.06%) experienced xerostomia or hyposalivation.

**Figure 1:**
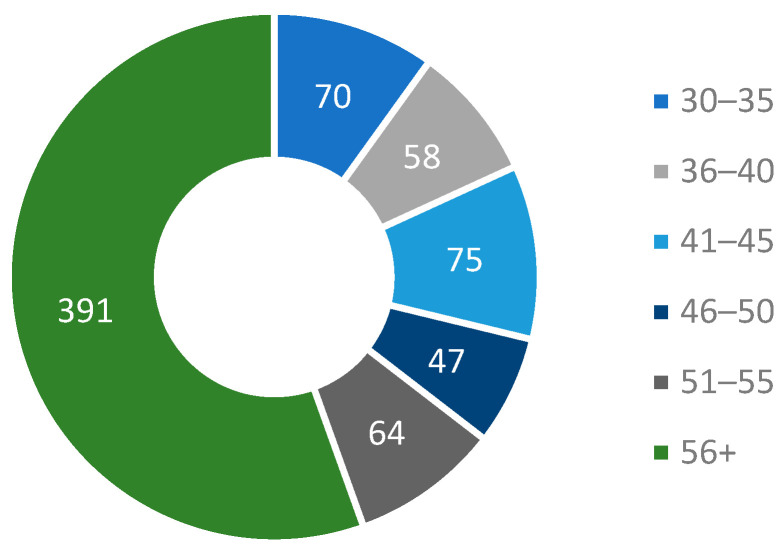
Age of patients experiencing xerostomia or hyposalivation.

The highest prevalence of hyposalivation (55.46%) was observed in patients aged 56 years and older (Figure 1).

Contributing risk factors which were evaluated across the entire sample population are detailed in Table 1. Marijuana use was less reported (7.75%) than other recreational drugs, such as alcohol (40.8%) and nicotine/tobacco (36.9%). Prescription medication use reported was high as well, with 68.8% of patients in the study taking some form of prescription medication.

Caries Risk Assessment (low, medium, or high) was separately evaluated in those patients with hyposalivation and those without, as listed in Table 2. A Chi-Square test of independence revealed a statistically significant association between hyposalivation status and caries risk, (df = 2, *n* = 1600, χ^2^ = 205.99, *p* < 0.001).

**Figure 2:**
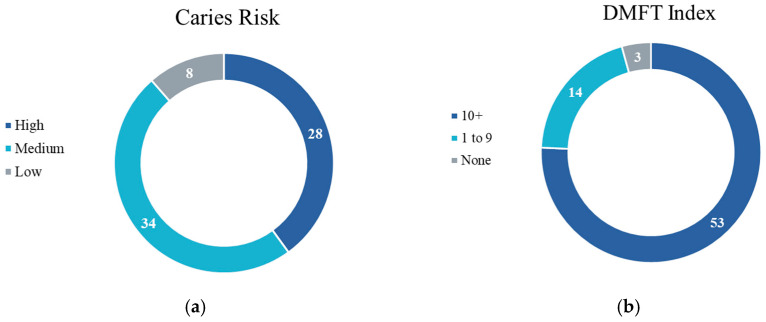
Additional information related to patients aged 30–35 years with hyposalivation: (**a**) Caries Risk Assessment of patients 30–35 years with hyposalivation; (**b**) DMFT index of patients 30–35 years with hyposalivation.

Caries risk was further investigated in younger patients that presented with hyposalivation to investigate declining dentition. Among patients aged 30–35 years old with hyposalivation, only 8 (11.4%) were determined to have low caries risk (Figure 2a). When highlighting the dentition status of these patients, Decayed, Missing, Filled Teeth (DMFT) indices were noted for each of these 70 patients, where it was found that 53 (76%) of these patients had a DMFT index of 10 or more, while only 3 (4%) of these patients had a DMFT index of 0 (Figure 2b).

In multivariate logistic regression analysis, marijuana use conferred the greatest risk of hyposalivation among all age groups, and was associated as an independent predictor of hyposalivation, as seen in Table 3a (RR = 3.10, OR = 1.70, *p* < 0.05). Among individuals aged 30–35 years, the risk of hyposalivation was especially elevated with the use of medications to address hypertension (OR = 3.05, *p* < 0.05) and mental health disorders (OR = 1.81, *p* < 0.05), as seen in Table 3b. No significant associations were found between other evaluated demographic or behavioral factors and the primary outcome.

## 4. Discussion

In this retrospective study of 705 patients afflicted with xerostomia or hyposalivation treated at UNLV Dental Clinics, it was found that marijuana use and medications addressing hypertension and mental health are increasingly associated with these symptoms. Additionally, patients aged 30–35 years with these symptoms exhibited more rapidly declining dentition compared to those without. These findings suggest that hyposalivation can predispose patients to a lifetime of oral health problems.

The findings are consistent with those of numerous studies that report similar trends in oral health outcomes in elderly dental patients [3,5,6]. However, unlike previous studies, the present study observed declining dentition associated with hyposalivation in a much younger population than seen before. This may be attributed to marijuana use and the increased prevalence of pharmacological interventions for cardiovascular and mental health disorders.

Several studies have reported on oral health considerations for those afflicted by hyposalivation, but few have analyzed the relationships between determinants of health and dentition status in a real-world setting using EHR data, particularly considering marijuana use as a contributing factor [24]. These results suggest that lack of salivation is an expanding clinical symptom increasingly seen in younger populations. This trend may indicate a need for better screening tools, a more thorough review of medical and social histories, and greater consideration of salivary flow in the context of oral health and dental treatments.

The present findings come into agreement with previous research that indicated there is a dramatic increase in the prevalence of older adults using cannabis warranting the investigation of both caries and tooth loss as a result of hyposalivation [22,24]. Others reported that habitual cannabis use has been correlated with an increased incidence of xerostomia, leukoedema, and a higher prevalence of Candida albicans infections [25,26,27]. Inconsistencies were noted between odds ratio and relative risk estimates in certain age groups, most notably within the 30–35-year subgroup, suggesting that while the relative risk associated with marijuana use is elevated overall, the clinical impact may vary by age group. These variations are likely attributable to smaller sample sizes within this specific cohort and age-related differences in exposure patterns, such as cumulative duration or frequency of cannabis use. Furthermore, limitations of this study include the absence of accurate or consistent measurements of marijuana use frequency or route of delivery, which may have influenced the observed associations. Such findings underscore the importance of interpreting relative measures of association alongside absolute risk differences to better contextualize the magnitude of clinical impact across demographic strata.

Understanding these patterns could help inform future clinical guidelines and commit awareness toward modifiable risk factors, such as marijuana use, which clinicians may not have previously considered. Additionally, the observed association between both hypertensive and mental health medications and hyposalivation reinforces the importance of reviewing medical history regularly. These insights may be valuable for clinicians and health systems aiming to improve dental health status in vulnerable populations.

This study has several strengths, including a large sample size, real-world data from routine clinical practice, and comprehensive EHR data extraction. However, it is important to acknowledge that EHRs may under-record non-medical drug use, as such information often depends on patient self-reporting and may not be systematically documented.

Despite these strengths, several limitations must be considered. As a retrospective study, inherent sources of bias and unmeasured confounding variables cannot be fully eliminated. Moreover, the retrospective design precludes the establishment of causal relationships; therefore, the observed associations should be interpreted with caution. In addition, due to the subjective nature of xerostomia, recall and detection biases may limit the number of patients reporting the sensation, meaning the true prevalence may be higher or lower than reported. Another limitation is the lack of detailed information regarding the methods of marijuana consumption, as different routes of administration may lead to varying durations and severities of hyposalivation. Furthermore, since marijuana legalization in Nevada was still in its early stages during the study period, self-report bias may have influenced documentation, with some patients being less likely to disclose marijuana use prior to legalization in 2017.

Despite these limitations, this study provides valuable insight into xerostomia and hyposalivation, particularly in the context of dental patients in Southern Nevada. Future research is warranted to validate these findings and explore their broader applicability across broader populations and clinical settings. Further investigations should also aim to obtain more detailed information regarding marijuana use, including the frequency, duration, and mode of consumption, as these factors may influence salivary gland function. Additionally, upcoming studies are planned to examine the specific classes of antihypertensive medications that may contribute to reduced salivary flow. A more comprehensive understanding of the pathophysiological mechanisms underlying hyposalivation and its associated risk factors could ultimately support the development of more effective preventive and therapeutic strategies in clinical practice.

The identification of hyposalivation and xerostomia risk in populations not classically associated with these symptoms highlights areas where targeted interventions or practice improvements may be warranted. This emphasizes the importance of meticulous and accurate medical and social history documentation. These results underscore the value of utilizing electronic health record data to inform evidence-based clinical decision-making and to stay ahead of changing social and medical environments in a more predictable fashion.

The present retrospective study analysis highlights the multifactorial nature of hyposalivation, underscoring the significant roles of marijuana use and common pharmacologic treatments, particularly for mental health and cardiovascular conditions. The observed association between marijuana use and subjective dry mouth is clinically relevant in the context of its expanding medical and recreational legalization across the United States. Similarly, the early and frequent use of medications with xerogenic potential may serve as an indicator for identifying individuals at increased risk for salivary gland hypofunction and subsequent oral health deterioration.

## 5. Conclusions

The present findings emphasize the importance of comprehensive medical and social history taking, early screening for salivary dysfunction, and patient education to mitigate downstream oral health consequences. Notably, hyposalivation and xerostomia are increasingly observed in younger adults, linked to pharmacological and behavioral factors.

## Figures and Tables

**Table 1:** Contributing risk factors evaluated for role in hyposalivation.

Risk Factor	Prevalence
Systemic Disease Presence	63.4%
Daily Prescription Medication Use	68.8%
Alcohol Use	40.8%
Nicotine/Tobacco Use	36.9%
Marijuana Use	7.75%

**Table 2:** Caries Risk Assessment of patients as related to their hyposalivation status.

Hyposalivation	Low Caries Risk	Medium Caries Risk	High Caries Risk
Yes (*n* = 705)	93 (13.2%)	233 (33.0%)	379 (53.8%)
No (*n* = 895)	171 (19.2%)	311 (34.7%)	413 (46.1%)

**Table 3:** (a) Patient predictors for hyposalivation (patients aged 30+). (b) Patient predictors for hyposalivation (patients aged 30–35).

**(a)**		
**Predictor**	**Odds Ratio (OR)** **[95% CI]**	**Relative Risk (RR)** **[95% CI]**
Systemic Disease Presence	1.20 [1.14, 1.26]	0.96 [0.91, 1.01]
Antihypertensive Medication Use	1.34 [1.28, 1.41]	0.96 [0.91, 1.01]
Antidepressant/Anti-anxiety Medication Use	1.22 [1.16, 1.28]	0.9 [0.86, 0.95]
Alcohol Use	0.90 [0.86, 0.95]	0.96 [0.91, 1.01]
Nicotine/Tobacco Use	1.40 [1.33, 1.47]	1.14 [1.09, 1.20]
Marijuana Use	1.70 * [1.62, 1.79]	3.10 * [2.95, 3.26]
**(b)**		
**Predictor**	**Odds Ratio (OR)** **[95% CI]**	**Relative Risk (RR)** **[95% CI]**
Systemic Disease Presence	1.10 [1.05, 1.16]	1.04 [0.99, 1.09]
Antihypertensive Medication Use	3.05 * [2.90, 3.20]	1.63 [1.55, 1.71]
Antidepressant/Anti-anxiety Medication Use	1.81 * [1.72, 1.90]	1.21 [1.15, 1.27]
Alcohol Use	1.00 [0.95, 1.05]	1.00 [0.95, 1.05]
Nicotine/Tobacco Use	1.10 [1.05, 1.16]	1.05 [1.00, 1.10]
Marijuana Use	1.10 [1.05, 1.16]	1.06 [1.01, 1.11]

(a) * Values found to be statistically significant at *p* < 0.05; sample size: *n* = 1600 patients. (b) * Values found to be statistically significant at *p* < 0.05; sample size: *n* = 70 patients.

## Data Availability

The data that support the findings of this study are available on request from the corresponding author. The data are not publicly available due to ethical restrictions.

## Abbreviations

The following abbreviations are used in this manuscript:

SSRI	Selective serotonin reuptake inhibitor
TCA	Tricyclic antidepressant
UNLV	University of Nevada, Las Vegas
EHR	Electronic health record
DMFT	Decayed, missing, filled teeth
OR	Odds ratio
RR	Relative risk
